# Adverse long-term outcomes following seizures in non-asphyxiated term infants: A population-based cohort study

**DOI:** 10.1038/s41390-025-04225-4

**Published:** 2025-07-05

**Authors:** Maria Jonsson, Lena Hellström-Westas, Per Wikman, Johan Ågren

**Affiliations:** Department of Women’s and Children’s Health, Uppsala University, Uppsala, Sweden

## Abstract

**Background:**

While birth asphyxia is a well-established cause of neonatal seizures associated with adverse outcome, less is known about outcomes after seizures in non-asphyxiated infants. We investigated adverse outcomes following neonatal seizures in infants born vigorous and without evidence of asphyxia.

**Methods:**

Swedish national cohort (2009–2015) including 656,088 live-born term vigorous infants. The exposure was a diagnosis of neonatal seizure. A combined adverse outcome included any diagnosis of cerebral palsy, epilepsy, intellectual and developmental disability, or death. Hazard ratios (HR) with 95% confidence intervals (CI) were calculated.

**Results:**

470 infants were diagnosed with seizures, and 299 (64%) had EEG-verified seizures. Among those with the combined adverse outcome, 75% were diagnosed the first year, corresponding to an incidence of 178 per 1000 of all infants and 224 per 1000 among EEG-verified cases. The one-year incidence rates were 220 per 1000 child years for all, and 283 per 1000 child years among those EEG-verified compared to 2.3 per 1000 child years without seizures. The HR for all seizures within 1 year was 95 (77–120), and for EEG-verified seizures 120 (96–160).

**Conclusion:**

This study demonstrates a strong association between neonatal seizures and adverse long-term outcome in vigorous term infants without asphyxia.

**Impact:**

- Term infants who were vigorous and non-asphyxiated at birth but later developed neonatal seizures faced a significant elevated risk of adverse outcomes, including death, cerebral palsy, epilepsy, intellectual and developmental disabilities.
- Among these adverse outcomes epilepsy was the most frequent and was predominantly diagnosed during the first year of life.
- Population-based studies utilizing national health and quality registers can greatly enhance our understanding of long-term outcomes following neonatal seizures.

## Background

Seizures are relatively common in newborn infants with a reported incidence of 1.0–3.5 per 1000 term neonates.^1–3^ The most prevalent etiologies of neonatal seizures include hypoxic ischemic encephalopathy (HIE), and perinatal stroke,^4^ with HIE accounting for approximately 50% of cases.^1,4,5^ Other significant but less frequent causes include intracranial infections, hypoglycemia, neonatal presentation of genetic epilepsies, inborn errors of metabolism, and congenital brain malformations.^6–8^

While neonatal seizures are associated with adverse outcomes such as death, cerebral palsy, epilepsy and intellectual and development disabilities, these outcomes are primarily linked to the underlying etiology rather than the seizures themselves. Moderate to severe HIE is a particularly strong risk factor for adverse outcome.^5,7,9,10^

Due to diagnostic challenges, several studies suggest that seizures confirmed by EEG are more strongly associated with adverse outcomes,^5,11,12^ however, this association was not verified in a recent randomized controlled trial involving infants with HIE.^13^ In studies that encompass all etiologies of seizures in term infants, the rates of adverse outcomes vary widely: cerebral palsy 4 to 46%, epilepsy 13 to 23%, and developmental delay 25 to 56%.^5,11,13–15^ Given that the high preponderance of seizures in infants with HIE to a great extent impact on these numbers, the reporting on long term outcomes of seizures in non-HIE cases remains limited.

National health registers have proven to be valuable resources for studying rare outcomes. In a large population-based study, we demonstrated that perinatal risk factors for neonatal seizures in vigorous non-asphyxiated term infants were similar to those observed in infants with moderate to severe HIE.^16^ In the present investigation, we aimed to clarify the long-term adverse outcomes following neonatal seizures in a national cohort of vigorous non-asphyxiated infants born at term.

## Methods

### Study design and population

**Fig. 1:**
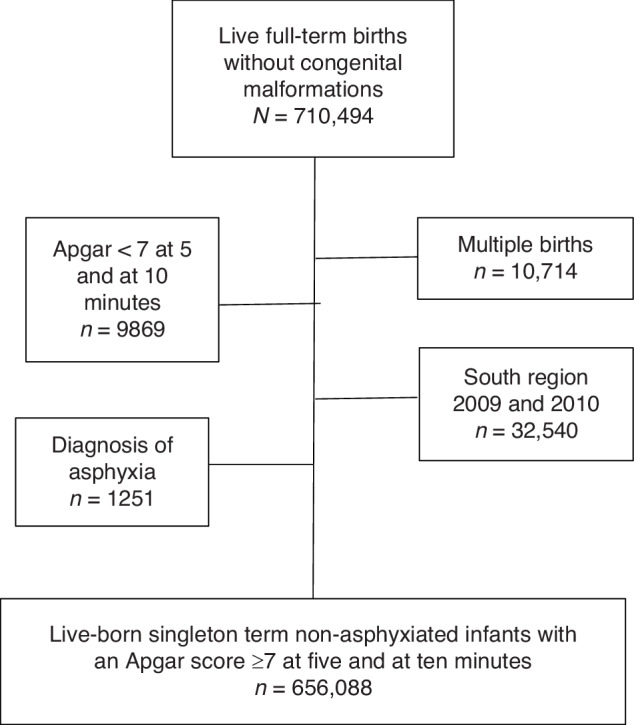
Flow chart of the study population.

This population-based cohort was derived from Swedish national health and quality registers covering the years 2009 to 2015. It included anonymized data from 710,494 term live-born singleton infants without congenital malformations. We excluded multiple births (*n* = 10,714), infants with Apgar score less than 7 at 5 and/or 10 min (*n* = 9,869), and infants with a diagnosis of birth asphyxia (P21, P91) according to the 10^th^ International Classification of Disease codes (ICD-10), including those that had received hypothermia treatment (*n* = 1,251). Additionally, because the southernmost region of Sweden did not submit data into the Swedish Neonatal Quality register (SNQ) for the years 2010 and 2011, infants (*n* = 32,540) from that region were excluded for those years. This left a final cohort of 656,088 liveborn term singleton non-asphyxiated infants (Fig. 1).

The study was approved by the regional ethics review board at Uppsala university in Uppsala Sweden (No 2015/156).

### Data sources

The national health registers used in this study were the Medical Birth Register (MBR), the National Patient register (NPR), and the Cause of death register, all held by the Swedish National Board of Health and Welfare.

The MBR was established in 1973 and has since then collected high quality data on more than 98% of births in Sweden.^17^ Registration of antenatal, obstetrical and neonatal care in the MBR is mandatory and done through standardized forms using check-boxes, numerical values, or ICD-codes. Data are collected prospectively and, after birth, both maternal and infant diagnoses are included. Although the validity of variables in the MBR is high and loss to follow-up negligible, the outcomes can be underestimated if diagnoses are incomplete or not registered.^17,18^

The NPR collects data on all inpatient care since 1987, and since 2001 it also includes data on all outpatient specialist care. Data have high validity and include primary diagnoses (ICD-codes), procedures, and length of stay, with 98% of medical records demonstrating correct coding when cross-validated with hospital notes, and holds a primary diagnosis in 99%.^19^

Although diagnostic variations exist between health-care regions, particularly among out-patient private health care providers, the registers’ reliability is considered excellent since specialized child health care and follow-up are uniformly provided within the public health care system.

Data were also retrieved from the national Swedish Neonatal Quality register (SNQ), which was established in 2001 and holds detailed information on neonatal care, and diagnoses for infants receiving care in a neonatal care unit within the first 28 days of life. SNQ data are prospectively collected with the use of standardized forms, and its agreement with other Swedish registers has proven excellent.^20^

All data were extracted and provided anonymized by The Swedish National Board of Health and Welfare, after linkage of data between the registers through use of the unique personal identification number provided to all Swedish citizens at birth.

Maternal body mass index (BMI; kg/m^2^) was calculated in early pregnancy and categorized according to the World Health Organization: underweight (BMI < 18.5), normal weight (BMI 18.5–24.9), overweight (BMI 25.0–29.9), and obesity (BMI ≥ 30.0). Maternal epilepsy was either self-reported at the first antenatal visit, or identified by current ICD-codes (G40, G41). Hypertension included chronic hypertension, gestational hypertension and preeclampsia. Diabetes mellitus included type 1 and 2, as well as gestational diabetes. Chronic hypertension and pregestational diabetes were identified by a ticked box in the antenatal record, or by an ICD diagnosis coded at discharge (O10, O139, O14, O24). Fever during labor (ICD-code O752), or chorioamnionitis (ICD-code O411) was used to define intrapartum infection. Mode of delivery was categorized as spontaneous vaginal, instrumental vaginal, or cesarean section. Small (SGA) and large for gestational age (LGA) were classified as a birthweight below, or above two standard deviations of the population mean weight for gestational age.^21^ A reassuring Apgar score was defined as having a score of equal to or above 7 at 5 and 10 min.^22^

### Exposure variable

The exposure was a neonatal seizure diagnosis (ICD-code P90), identified in the MBR or SNQ. Seizures were classified as EEG-verified or clinical, according to a tick box (“yes”) for EEG in the SNQ form.^23^ According to the instructions in the register, amplitude-integrated electroencephalography (aEEG) or conventional EEG-verified seizures denote that seizures were identified in a recording using aEEG/EEG. However, the tick-box does not provide further clinical information. The use of aEEG monitoring and/or repeated conventional EEGs are mandated by the Swedish Neonatal Society when there are clinically suspected seizures, or other signs of encephalopathy.

The diagnosis of seizures most commonly relies on a combination of history, clinical symptoms and aEEG or conventional EEG/video-EEG assessed by neurophysiologists.

A validation of SNQ data demonstrated that when compared to EEG, aEEG had a positive predictive value of 90% for the seizure diagnosis (P90.9 A).^23^

To explore underlying causes of seizures, potential associated morbidities were investigated by identifying the ICD-codes from MBR, NPR, or SNQ, including infection in the central nervous system (P35-P37), neonatal ischemic or hemorrhagic stroke (P91.0D, P52, P10), hypoglycemia <2.6 mmol/L beyond 3 h of age (P70), metabolic disorders (P72-P74), withdrawal symptoms (P96.1, P96.2), and kernicterus (P57).

### Outcome

The pre-defined combined adverse outcome variable included any diagnosis in the NPR of epilepsy (G40, G 41), cerebral palsy (G80- G83), intellectual and developmental disabilities [IDD, (F70-F79, F80-F89)], or death in the Cause of Death register. These adverse outcomes were also considered separately.

### Statistical analyses

Descriptive statistics are presented as counts and percentages for categorical variables and as means ± standard deviations (SD) for continuous variables. Person-time incidence rates were calculated for the outcomes by dividing the number of events by total person-time, expressed as events per 1000 person-years.

Time-to-event data, specifically the time to outcome, were visualized using the Kaplan-Meier product limit estimator. The log-rank test was used to compare survival curves for infants with and without seizures. We employed Cox proportional hazards regression models to estimate hazard ratios (HRs) and 95% confidence intervals (CI), quantifying the relative risk of seizures associated with covariates over the study period, 2009 to 2015. Since all births during this period were included, this resulted in variable ages at the time of data extraction. Half of the infants were younger than three years while the other half was older than three years at follow-up. Proportional hazards assumptions were assessed by graphical inspection of scaled Schoenfeld residuals. The Cox model assumption of proportional hazards was not fulfilled for the entire study period, rather we found the underlying HRs for exposed infants to be comparatively higher during the first year of life, followed by gradually lower HRs. To address this, separate regressions models were applied up to and after 1 year. This ensures that hazards are proportional within each interval and also enables more nuanced analysis than what could have been achieved by applying any singular model to the entire study period.

Confounders were selected based on clinical relevance, and prior literature.^16,24^ The first model included antenatal confounders: parity, BMI, hypertensive disorders, and diabetes mellitus. The second model included labor and delivery-related confounders such as intrapartum infection, mode of delivery, and birthweight classifications (SGA and LGA) and possible seizure etiologies: infection in the central nervous system, stroke, hypoglycemia, metabolic disorders withdrawal symptoms and kernicterus. Confounders were entered into the Cox model as fixed covariates to assess their impact on the outcome of interest.

Cases with unchecked boxes or a missing ICD code were assumed not to have the condition. Missing data were present for maternal age, BMI, mode of delivery, and birthweight, and were managed by multiple imputations by chained equations using the Multivariate Imputation by Chained Equations package in R.

Time to event was measured in days from birth to the first diagnosis, with follow-up ending at the time of data extraction (December 31, 2015) or death. For the combined adverse outcome, time was tracked in days from birth until the earliest occurrence of any component event until end of data extraction, or death. For infants with multiple diagnoses, the time-to-event analysis was based on the earliest diagnosis. IBM SPSS Statistics version 28.0 and R 4.3.3 were used for statistical analyses.

## Results

Out of 656,088 live-born, singleton, term infants without asphyxia, 470 (0.72 per 1000) were diagnosed with neonatal seizures. Among these, 299 (64%) were confirmed using aEEG/EEG. Table 1 presents pregnancy and labor characteristics in the entire study population categorized into infants without seizures and those with seizures (both clinically diagnosed and aEEG/EEG-verified). Mothers of infants with seizures had higher BMI, were more often primiparous, and had higher rates of hypertensive disorders, and diabetes. Infants with seizures were also more often born post term, exposed to infection during labor (3.4% vs. 0.9%) and delivered by instrumental vaginal delivery, and cesarean section.

**Table 1:** Pregnancy and labor characteristics of the entire study population, categorized into no seizures and all seizures (i.e. clinical and aEEG/EEG-verified).

	No seizures *N* = 655 618	All seizures *N* = 470	*P-*value
	*n* (%)	*n* (%)	
Age (years in mean ± SD)	30.3 ± 5.2	30.3 ± 5.3	0.94
missing	2	0	
Primiparous	283 769 (43.3)	272 (58.0)	<0.001
BMI (kg/m^2^)^a^			<0.001
<18.5	15 549 (2.5)	9 (2.0)	
18.5–24.9	363 885 (58.9)	223 (50.3)	
25–29.9	160 346 (26.0)	128 (28.9)	
30–34.9	54 954 (8.9)	54 (12.2)	
35–39.9	17 045 (2.8)	21 (4.7)	
≥ 40	5 966 (1.0)	8 (1.8)	
missing	37 873 (5.8)	27 (5.8)	
Epilepsy^b^	3 259 (0.5)	4 (0.9)	0.27
Hypertension^c^	25 338 (3.9)	51 (10.9)	<0.001
Diabetes mellitus^d^	9 999 (1.5)	22 (4.7)	<0.001
Gestational age at birth (weeks)			0.002
37–40	482 803 (73.6)	326 (69.4)	
41 (late term)	125 971 (19.2)	91 (19.4)	
≥ 42 (post term)	46 844 (7.1)	53 (11.3)	
Intrapartum infection^e^	5 833 (0.9)	16 (3.4)	<0.001
Mode of delivery			<0.001
spontaneous vaginal	502 933 (77.7)	269 (57.8)	
instrumental^f^	44 105 (6.8)	70 (15.1)	
cesarean section	100 014 (15.5)	126 (27.1)	
missing	8566 (1.3)	5 (1.1)	
Female sex	321 348 (49.0)	210 (44.7)	0.06
Birthweight (grams)	3568 ± 504	3592 ± 486	0.31
missing	703 (0.1)	1 (0.2)	
Large for gestational age	22 232 (3.4)	27 (5.7)	<0.001
Small for gestational age	11 763 (1.8)	21 (4.5)	<0.001

*n* numbers.

^a^Body mass index; calculated from self-reported height and measured weight at first antenatal visit (gestational week 8–10).

^b^Self-reported at first antenatal visit and/or a diagnosis of epilepsy in the Swedish Medical Birth Register.

^c^Chronic hypertension, gestational hypertension, preeclampsia.

^d^Diabetes mellitus type 1 and 2, gestational diabetes mellitus,

^e^Chorioamnionitis and/or fever during labor.

^f^Vacuum extraction or forceps.

Among seizure etiologies neonatal stroke (19.6%), and hypoglycemia (18.1%) were the most frequently diagnosed (Table 2). Central nervous system infection and disorders of metabolism also accounted for a significant proportion of cases, while withdrawal symptoms and kernicterus were extremely infrequent.

**Table 2:** Morbidities in the neonatal period in infants with and without neonatal seizures.

	No neonatal seizures *N* = 655 618	All seizures *N* = 470	EEG verified seizures, *N* = 299
Infection in the central nervous system	54 (0.0)	11 (2.3)	6 (2.0)
Neonatal stroke^1^	53 (0.0)	92 (19.6)	67 (22.4)
Hypoglycemia^2^	4 753 (0.7)	85 (18.1)	46 (15.4)
Metabolism disorder	210 (0.8)	18 (3.8)	11 (3.7)
Withdrawal symptoms	163 (0.0)	3 (0.6)	1 (0.6)
Kernicterus	38 (0.0)	1 (0.2)	1 (0.6)

Numbers are presented as *n* (%).

^1^ischemic or hemorrhagic stroke.

^2^< 2.6 mmol/L after 3 h.

The mean follow-up duration, until an outcome diagnosis was recorded, or until the conclusion of the data retrieval period, was 2.9 years for infants with seizures and 3.3 years for those without seizures. In total, 112 infants experienced the combined outcome, equating to 238 per 1000 cases overall. Among those with aEEG/EEG-verified seizures the incidence was 294 per 1000 of compared to 10 per 1000 among those without seizure (i.e. no clinical or aEEG-verified seizures; data not shown in table).

Table 3 presents the results including all seizures with the incidences and HRs for outcomes occurring within one year of age, and from one year onward. For infants under one year, the incidence of the combined adverse outcome associated with all seizures was 178 per 1000 with an incidence rate of 220 per 1000 child- years. Infants without seizures had an incidence of 2.12 per 1000 cases resulting in a crude HR of 95 (95% CI 77, 120), and decreasing to a HR of 77 (95% CI 60, 99) after the adjustments in Model 2. Beyond one year of age, the combined adverse outcome was observed in 84 per 1000 newborns with seizures with an incidence rate of 22 per 1000 child-years. The corresponding figures for infants without seizures were 9.48 per 1,000 births and an incidence rate of 2.5 per 1000 child-years, yielding a HR of 9.3 (95% CI 6.4, 13), which decreased to 7.6 (95% CI 5.2, 11) after the adjustments in Model 2. Among the individual outcomes, epilepsy was the most prevalent and primarily occurred within the first year of life in infants with seizures at a rate of 121/1000 infants compared to 1.1/1000 in those without seizures. This corresponded to a HR of 130 (95% CI 97, 170), which decreased to 110 (95% CI 79, 150) after the adjustments in Model 2.

**Table 3:** The outcomes for infants with and without neonatal seizures are presented with hazard ratios for two age groups: under one year and one year or older.

	No seizures, *N* = 655 618				All seizures, *N* = 470				Hazard Ratio (95% CI)		
Outcome	*n*	1/1000 births	Child-years of follow-up	Rate /1000 child- years	*n*	1/1000 births	Child-years of follow-up	Rate /1000 child-years	Crude	Adjusted^a^	Adjusted^b^
Epilepsy <1 year	696	1.1	605 243	1.1	57	121	387	147	130 (97-170)	120 (94-160)	110 (79-150)
≥1 year	1102	1.9	2 145 606	0.5	11	32	1316	8.3	16 (9-30)	16 (8.8-29)	14 (7.8-26)
Cerebral palsy <1 year	158	0.2	605 534	0.3	12	25	421	28	110 (61-120)	110 (61-200)	82 (43-160)
≥1 year	346	0.6	2 148 761	0.2	13	34	1449	8.9	56 (32-97)	56 (32-97)	42 (23-78)
IDD <1 year	313	0.5	605 494	0.5	11	23	420	26.2	51 (28-93)	48 (26-88)	43 (23-80)
≥1 year	4 137	7.4	2 142 468	1.9	24	63	1449	16.5	8.7 (5.8-13)	8.3 (5.6-12)	7.2 (4.7-11)
Death <1 year	311	0.5	605 584	0.5	15	32	424	35	69 (41-120)	70 (42-120)	41 (21-79)
≥1 year	142	0.3	2 150 163	0.07	1	2.6	1510	0.6	10 (1.4-71)	10 (1.4-73)	6.3 (0.86-47)
Combined^c^ <1year	1 392	2.12	605 125	2.3	84	178	382	220	95 (77-120)	91 (73–110)	77 (60-99)
≥1 year	5 250	9.48	2 137 592	2.5	28	84	1251	22	9.3 (6.4-13)	9 (6.2-13)	7.6 (5.2-11)

*IDD* Intellectual and Development Disabilities, *n* numbers, *HR* hazard ratio *CI* confidence interval.

^a^Adjusted for parity, body mass index, hypertension and diabetes mellitus.

^b^Also adjusted för SGA, LGA, intrapartum infection, mode of delivery and etiologies. For epilepsy, adjustment for maternal epilepsy was added.

^c^Combined outcome; any of cerebral palsy, epilepsy, IDD and death. The combined outcome was adjusted for maternal epilepsy.

Table 4 presents infants with aEEG/EEG-verified neonatal seizures and their outcomes within the first year, and from one year onward. The combined adverse outcome, both as regards to incidence and incidence rate, was notably higher with aEEG/EEG verified seizures than among those with no seizures resulting in a HR of 120 (95% CI 96, 160) for infants under one year of age. After adjustments in Model 2, this decreased to 100 (95% CI, 93,190). Among the individual outcomes, epilepsy was the most common for infants in this age group, with an HR of 160 (120, 220) which decreased to 130 (95% CI, 93, 190) after adjustments.

**Table 4:** The outcomes for infants without neonatal seizures and those with aEEG/EEG-verified seizures are presented with hazard ratios for two age groups: under one year and one year or older.

	No seizures, *N* = 655 618				aEEG/EEG verified seizures, *N* = 299				Hazard Ratio (95% CI)		
Outcome	*n*	1/1000 births	Child-years of follow-up	Rate /1000 child- years	*n*	1/1000 births	Child-years of follow-up	Rate /1000 child-years	Crude	Adjusted^a^	Adjusted^b^
Epilepsy <1 year	696	1.06	605 242	1.1	45	150	240	187	160 (120-220)	150 (110-210)	130 (93-190)
≥1 year	1 102	1.9	2 145 606	0.5	8	38	811	9.8	19 (9.5-38)	19 (9.3-37)	17 (8.1-34)
Cerebral palsy <1 year	158	0.2	605 534	0.3	9	30	268	33.5	130 (67-250)	130 (66-250)	96 (45-200)
≥1 year	346	0.6	2 148 761	0.2	12	50	915	13.1	81 (46-140)	81 (45-140)	60 (32-120)
IDD <1 year	313	0.5	605 494	0.5	9	30.1	267	33.6	66 (34-130)	63 (32-120)	57 (28-110)
≥1 year	4137	7.5	2 142 468	1.9	19	79.8	920	20.6	11 (6.8-17)	10 (6.6-16)	9.3 (5.7-15)
Death <1 year	311	0.5	605 584	0.5	11	37	270	41	79 (43-140)	78 (43-140)	44 (20-100)
≥1 year	142	0.3	2 150 163	0.1	0	0	965	-	-	-	-
Combined^c^ <1 year	1 392	2.1	605 125	2.3	67	224	236	283	120 (96-160)	120 (91-150)	100 (77-130)
≥1 year	5 250	9.5	2 137 592	2.5	21	105	757	27	11 (7.4-17)	11 (7.2-17)	9.7 (6.3-15)

*IDD* Intellectual and Development Disabilities, *n* numbers, *HR* hazard ratio, *CI* confidence interval.

^a^Adjusted for parity, body mass index, hypertension and diabetes mellitus.

^b^Also adjusted för SGA, LGA, intrapartum infection mode of delivery and etiologies. For the composite outcome and epilepsy, maternal epilepsy was added.

^c^Combined; any outcome of cerebral palsy, epilepsy, IDD and death.

**Fig. 2:**
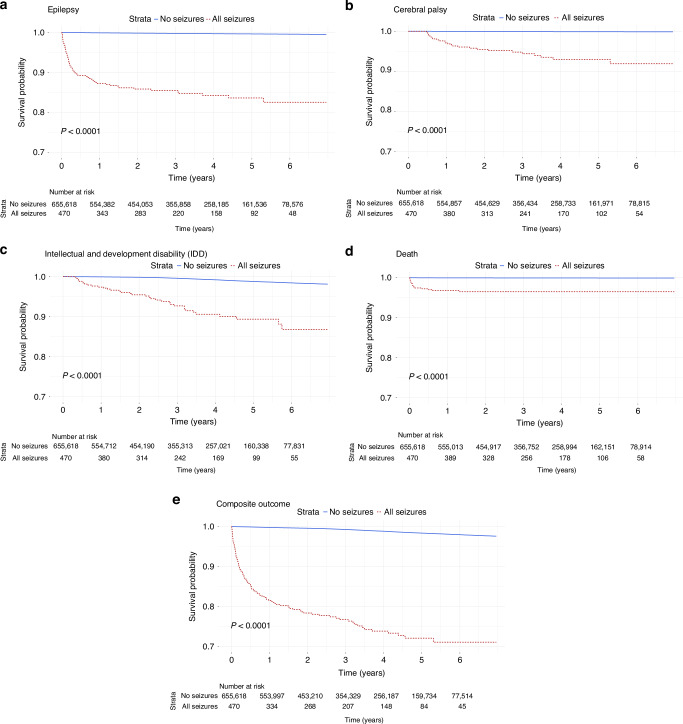
Kaplan-Meier (KM) analyses. Figure 2a to 2c show KM curves for the adverse outcomes. Included are the numbers at risk and the result of the log-rank test. Time to event is presented in years (x-axis). : **a** Epilepsy; **b** Cerebral palsy; **c** intellectual and developmental disabilities (IDD); **d** Mortality; **e** All outcomes/combined adverse outcomes.

Figure 2a–e illustrate Kaplan-Meier analyses, showing the estimated percentages of infants experiencing the combined and individual adverse outcomes at various time-points (in years) after birth. Within the first year, nearly 20% of infants with neonatal seizures experienced the combined outcome. Most deaths occurred within the first six months (Fig. 2d). The reported causes of death (*n* = 11) among infants with neonatal seizures were: respiratory failure (*n* = 4), cardiac dysrhythmia (*n* = 3), and status epilepticus, cerebral infarction, congenital herpes virus infection, unknown (all *n* = 1). The log-rank test showed statistically significant differences in survival curves between infants with and without seizures, both for the combined adverse outcome and the individual outcomes (*p* < 0.0001).

## Discussion

This large population-based study of vigorous non-asphyxiated term infants found a strong association between neonatal seizures and adverse long-term outcome. Approximately one fourth of infants with neonatal seizures experienced the combined adverse outcome, facing a risk more than ninety times higher during the first year compared to infants without seizures. Epilepsy and cerebral palsy exhibited the highest hazard ratios, and these associations were further strengthened when the risk calculations were based on seizures confirmed by aEEG/EEG.

### Strengths

The population-based design and the substantial size of our cohort are significant strengths that allowed us to investigate relatively rare outcomes while accounting for multiple covariates. To the best of our knowledge, this is the largest study conducted to date on outcomes following neonatal seizures. The diagnoses for both the exposure and outcomes were sourced from national health and quality registers, which provide high coverage and strong internal validity further enhancing the robustness of our findings.^17,20,23^ Additionally, we report outcomes associated with neonatal seizures diagnosed both clinically, and verified by aEEG/EEG.

### Limitations

One notable limitation of this study is the time to follow-up and data extraction, as the outcomes investigated could manifest later in childhood. Consequently, the associated risks following neonatal seizures could be higher than those reported. Additionally, the register-based study design restricts our ability to analyze the associations between individual data points and the severity of impairments. However, the Kaplan-Meier diagrams clearly illustrate that the outcomes, - except for intellectual and developmental disabilities which tends to be diagnosed later - primarily occur during the first few years of life. Furthermore, we recognize that the use of ICD-codes for outcome classification only provides a crude representation of illness severity. Although we lacked information on the timing of seizures within the neonatal period, it is important to note that most seizures in term neonates typically occur within the first three days of life. Therefore, our findings should be interpreted as relevant to seizures that present early after birth.^5^ Due to limited number of variables in the registers, we were unable to further analyze individual confounding factors of importance, such as genetic testing results for infants.

### Interpretation

While there is substantial evidence indicating that neonatal seizures, regardless of their underlying etiology, are significant predictors of subsequent adverse outcomes, including mortality, neurodevelopmental impairment and epilepsy,^7,10,25^ these associations have primarily been demonstrated in relation to HIE.^7,26–28^ Outcomes associated with other causes of neonatal seizures have not been extensively explored.^25^ Comparing studies on long-term outcomes after neonatal seizures is challenging due to variations in study designs, sample sizes, inclusion of both preterm and term births, differing diagnostic criteria and discrepancies in follow-up duration. A review of studies from the last two decades, involving more than 100 subjects (all retrospective single center cohorts), revealed considerable variability in the proportions of unfavorable outcomes: mortality rates ranged from 7% to 38%, epilepsy 6% to 27%, cerebral palsy 4% to 25% and intellectual disability from 25% to 48%.^5,9,11,29^ In a population-based study by Ronen et al., which included 62 term infants with neonatal seizures, it was found that 29% developed epilepsy, 25% had cerebral palsy, 25% experienced intellectual disability, and 16% had died within a follow-up period of 10 years.^7^ In that study, which also included cases of HIE, the risk for developing epilepsy was found to be increased forty times compared to the general population. In contrast, our present study uniquely focuses on outcomes following neonatal seizures in non-asphyxiated infants.

We believe that the exceptionally large sample of prospectively collected population-based data in the present investigation provides robust results for this specific subgroup. The proportion of infants with neonatal seizures who later developed epilepsy aligns with previous reports; however, the proportions of infants affected by cerebral palsy, intellectual and developmental disabilities, and mortality were lower. Consistent with previous studies, the current time-to-event analyses indicate that the onset of epilepsy predominantly occurs within the first year of life.^7,27,28^

While neonatal seizures are often identified based on clinical symptoms, their accurate diagnosis is significantly enhanced when confirmed by EEG. Studies have shown that the rate of adverse outcomes is higher when seizures are EEG-confirmed.^27,30–32^ It has been suggested that excluding clinically diagnosed seizures may introduce a selection bias favoring more severely affected infants in highly specialized care units, which may not accurately reflect real-world clinical practice.^7^ In the current study we report aEEG/EEG verified seizures separately. This does not mean that the other cases did not have an EEG recorded, since Sweden from 2006 has national guidelines that strongly mandates neurophysiological monitoring for infants with suspected seizures. While we, from our data, cannot report how the individual cases received their seizure diagnosis, it is thus possible that some infants with a low seizure burden were still deemed ``not verified by EEG/aEEG.” Nevertheless, the fact that seizures stated as verified by aEEG/EEG were associated with higher incidences and hazard ratios for all outcomes, clearly demonstrates that greater diagnostic precision (or higher seizure burden) enhances prognostic accuracy.

Understanding the specific etiology of neonatal seizures is likely crucial, as some causes (e.g., severe/prolonged hypoglycemia and kernicterus) may be preventable. Conversely, conditions such as neonatal stroke might be more challenging to address in otherwise well-appearing newborns. Previous studies have shown that 30–50% of infants with moderate HIE and over 90% with severe HIE experience adverse long-term outcomes.^33^ Our findings indicate that while the risk of unfavorable long-term outcomes after neonatal seizures in a non-asphyxiated newborn infant is lower than that observed in infants with moderate to severe HIE it remains significant and aligns with outcomes reported after mild HIE.^34^

Epidemiological studies like ours can identify associations between exposures and outcomes, as well as relationships to potential contributing factors. To facilitate early identification of cases and to tailor interventions that might improve long-term neurological outcomes, future investigations into outcomes following neonatal seizures should ideally include more detailed exposure data and comprehensive neurodevelopmental follow-up. We believe that national health registers are valuable tools for conducting population-based studies on long-term outcomes following neonatal seizures.

## Conclusions

Term infants who are vigorous and non-asphyxiated at birth but present with seizures during the neonatal period face a significantly associated risk of adverse neurodevelopment outcomes and mortality. While accurate prediction of long-term outcome in an individual infant remains challenging, data from this cohort can serve as a valuable baseline for comparison in future studies. Along with insights into seizure etiology, these findings provide crucial information for neonatal care providers, which may influence individual follow-up planning and parental counseling.

## Data Availability

The data in this study cannot be made publicly available due to Swedish legal restrictions that prohibit the disclosure of register-based data.
